# Individualized Skill-Based Manikin Training Coupled with a Team Approach May Enhance Delivery Room Neonatal Resuscitation in Low-Resource Settings

**DOI:** 10.3390/children13050679

**Published:** 2026-05-15

**Authors:** Emily Ahn, Jackline Cypriane, Nyemo Peter, Ester Ngowi, Aisa Shayo, Pendo Mlay, Jeffrey Perlman

**Affiliations:** 1 Division of Neonatology, NewYork-Presbyterian-Weill Cornell Medicine, New York, NY 10065, USA; 2 Department of Obstetrics and Gynecology, Kilimanjaro Christian Medical Centre, Moshi 251, Tanzania; 3 Department of Pediatrics, Kilimanjaro Christian Medical Centre, Moshi 251, Tanzania; 4 Dean School of Medicine, KCMC University, Moshi 251, Tanzania

**Keywords:** global health, neonatal resuscitation training, teamwork

## Abstract

**Highlights:**

**What are the main findings?**

- When performing individual bag mask ventilation testing, midwives more often demonstrated poor performance, often related to low inflating pressures presumed to be secondary to a mask leak.
- Clinical observations post-team training revealed that effective bag mask ventilation was achieved in most observed resuscitations, as evidenced by chest rise and an increase in heart rate.

**What are the implications of the main findings?**

- A multi-modal approach that combines individual skills with team simulation training may enhance neonatal resuscitation in low-resource settings.
- The addition of team training appears to be associated with effective bag mask ventilation in most observed cases.

**Abstract:**

**Objectives**: We aimed to implement a multi-modal resuscitation curriculum that first focuses on mastering individual skills, followed by team communication training, and study its effect on adherence to neonatal resuscitation steps, including effective bag mask ventilation (BMV) during high-risk deliveries. **Methods**: This was a single-center prospective observational manikin study conducted in a low-resource regional referral hospital. It included three phases: 1. pre-assessment; 2. individual BMV skill assessment followed by BMV skill testing; and 3. team training with simulation practice. Clinical observations of high-risk deliveries followed. **Results:** During the pre-assessment, midwives and residents knew when to start BMV but not the parameters for efficacious BMV (such as rate and inflation pressure). Midwives initially outperformed residents in individualized manikin assessments with 88% vs. 27%, *p* < 0.01, respectively, demonstrating efficacious BMV on the first attempt. However, on BMV testing, midwives more often demonstrated poor BMV performance, as evidenced by a persistent heart rate <100 bpm (31% vs. 8%, *p* = 0.04, respectively). Ineffective BMV in both groups was often related to low inflation pressures presumed to be in part secondary to a mask leak. Clinical observations demonstrated close adherence to pre-delivery preparation, basic neonatal stabilization, and resuscitation interventions. BMV was effective in most observed resuscitations as evidenced by chest rise and an increase in heart rate. Debriefing was the least performed team-training skill. **Conclusions**: A multi-modal approach combining individual skills training with team training that focuses on preparation, communication, and immediate skill feedback may enhance neonatal resuscitation in low-resource settings.

## 1. Introduction

Neonatal resuscitation training in a low-resource setting (LRS) has often been taught using the World Health Organization’s Essential Newborn Care Course [1] or the American Academy of Pediatrics’ (AAP) Helping Babies Breathe (HBB) [2]. These programs have been effective in reducing early neonatal mortality [3] and/or still births [4,5]. However, a decline in bag mask ventilation (BMV) skills has been seen over time [6]. The above courses have focused on individual training using low-cost manikins, which is appropriate in facilities where human resources are limited. By contrast, in larger hospitals with high-risk deliveries and more human resources, the presence of a team may be justified. In support of this, the International Liaison Committee on Resuscitation (ILCOR) recommends teaching teamwork during life support training [7].

BMV is the cornerstone of effective resuscitation in a depressed neonate. Its efficacy is dependent on delivery of an adequate rate, sufficient pressure, proper head positioning, and avoiding mask leaks. Administering effective BMV in the LRS is complex, influenced in part by training, frequency of BMV application, and equipment that often lacks pressure monitoring. Historically, at Kilimanjaro Christian Medical Centre (KCMC), a referral hospital in Northern Tanzania, the midwife was the sole provider for post-delivery neonatal care with occasional help from pediatric healthcare professionals, i.e., pediatric residents and, on occasion, pediatric consultants. Midwives and pediatric residents are trained in HBB, and pediatric residents receive additional training in advanced pediatric resuscitation while on ward rotations. Tanzania has national guidelines for delivery room resuscitation that recommend following the HBB algorithm and suggest considering advanced resuscitation in institutions where three healthcare professionals are available with enough skill and experience, as well as necessary equipment in the delivery room (adrenaline) [8].

We hypothesized that providing a multi-modal resuscitation curriculum that first focuses on mastering individual skills, followed by team communication training, would translate to adherence to neonatal resuscitation steps with effective BMV during high-risk deliveries. Our objectives were to 1. identify and correct suboptimal BMV techniques with repeated individual manikin training; 2. implement team communication training that focuses on anticipation, preparation, stabilization, and resuscitation; and 3. measure the impact of this training through clinical observation of high-risk deliveries.

## 2. Materials and Methods

### 2.1. Study Setting

This study was conducted at KCMC, a regional referral and teaching hospital in the LRS. Delivery room (DR) resuscitation is conducted primarily by midwives with occasional support from pediatric residents. If additional help is anticipated, midwives contact the assigned resident via cell phone. All midwives and pediatric residents are trained in HBB and follow its neonatal resuscitation algorithm. Pediatric residents often receive additional training in advanced pediatric resuscitation while on other rotations. For advanced resuscitation, national guidelines are followed as discussed above. As a referral hospital, KCMC delivers both low- and high-risk cases. Infants requiring resuscitation after birth are brought to a separate room, which contains three radiant warmers, resuscitation equipment, pulse oximeters, and dry electrode ECG devices. BMV is performed using self-inflating resuscitators that do not have a manometer to measure inflating pressure or the capacity to provide end-expiratory pressure. Continuous positive airway pressure and the ability to intubate are unavailable in the resuscitation room.

### 2.2. Study Protocol

**Figure 1:**
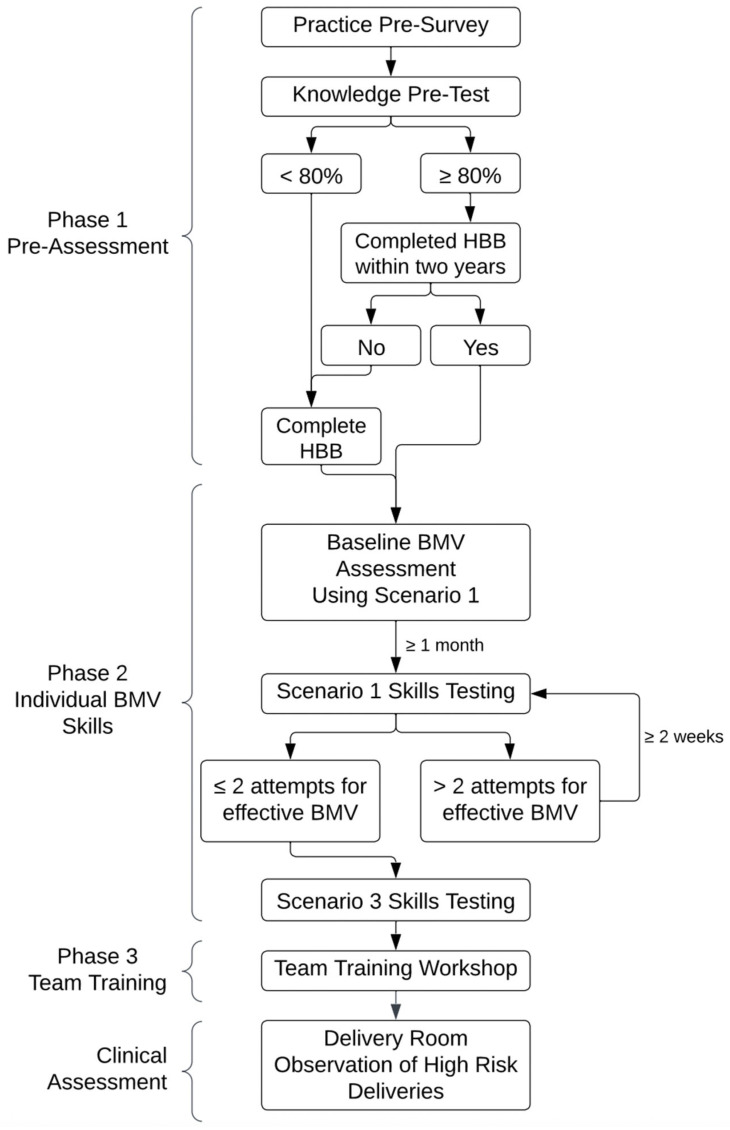
Flow diagram by study phase: pre-assessment, individual BMV skills, and team training. Effective BMV was defined as obtaining nonobstructive head positioning, achieving chest rise, ventilation rate of 40–60 bpm, inflating pressure of 20–30 mBar, and a rising heart rate. After completion of the study, a research assistant observed high-risk deliveries.

This was a prospective observational manikin study that evaluated the impact of multi-modal resuscitation training on preparedness and adherence to neonatal resuscitation guidelines, including the effectiveness of BMV. The training included three phases: 1. pre-assessment of experience and knowledge in resuscitation; 2. individual BMV skill assessments followed by individual BMV skill testing; and 3. team training with simulation practice (Figure 1). Assessment included an evaluation of the participants’ knowledge or skills prior to any feedback/training, whereas testing was a measure of BMV skill retention. This was followed by clinical observations of high-risk deliveries.

A midwife and physician were identified as study champions to locally oversee the implementation of each phase. Champions obtained verbal consent from participants and monitored all BMV sessions. Eligible participants included labor and delivery midwives and pediatric residents. Phase data was collected from June 2023 to April 2024.

#### 2.2.1. Phase 1: Pre-Assessment

All participants completed a practice pre-survey and knowledge pre-test (Supplementary Materials). The practice survey focused on recent BMV experience. The multiple-choice knowledge test focused on steps of resuscitation. Questions were adapted from the HBB knowledge test [9] with additional questions related to BMV parameters. Participants who had not completed an HBB course in the past two years and/or who scored <80% on the knowledge test were required to complete an HBB course prior to starting Phase two.

#### 2.2.2. Phase 2: Individual BMV Skills

##### NeoNatalie Live Trainer

**Figure 2:**
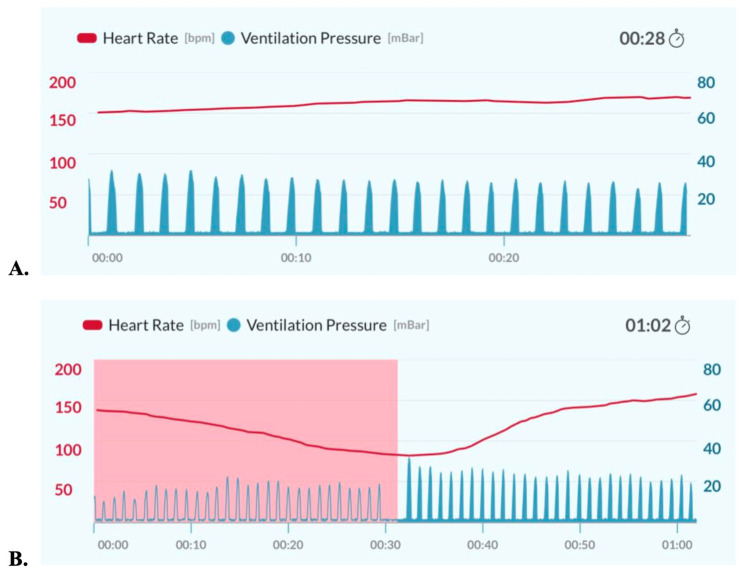
Examples of NeoNatalie Live feedback with (**A**) effective and (**B**) ineffective ventilation. Blue lines represent ventilation rate and inflating pressure; the red line represents HR; and pink shading represents obstructive head positioning. (**A**). With effective ventilation, the HR remains >150 bpm. (**B**). Airway obstruction results in ineffective ventilation and HR < 100 bpm.

All BMV sessions utilized the NeoNatalie Live trainer (Laerdal Global Health, Stavanger, Norway) [10], which was paired with a NeoBeat (Laerdal Global Health, Stavanger, Norway) [11] and a dry electrode ECG monitor that displays the trainer’s heart rate (HR). The NeoNatalie Live trainer is a smart manikin that can sense resuscitative actions and adjust the manikin’s HR based on ventilation efficacy. It has four preset scenarios of varying difficulty. Scenario One and Scenario Three were utilized in this study. Scenario One simulates the apneic delivery of an infant with a normal HR and “normal lung compliance”. Scenario Three simulates an apneic infant with a HR < 100 bpm and “low lung compliance”. This scenario required ventilation corrective steps, i.e., increasing inflation pressure, to achieve a visible chest rise and an increase in HR. The trainer was used to measure ventilation rate, inflating pressure, obstructive head positioning, and HR. This data was blinded during active ventilation attempts but available for immediate feedback and pictorially displayed on a tablet for review by study champions and participants after the ventilation attempt was complete (Figure 2). All participants were oriented to the trainer at the start of each scenario. Participants were informed that initial resuscitative steps had been completed (drying and stimulation) and to begin ventilation for apnea.

##### BMV Assessment

Participants first underwent a baseline BMV assessment utilizing Scenario One. After initial assessment, participants practiced this scenario coupled with champion teaching/feedback (rapid cycle deliberate practice) until effective BMV was achieved once. Effective BMV was defined as obtaining nonobstructive head positioning, delivering breaths at a rate of 40–60 bpm with an inflating pressure of 20–30 mBar (equivalent to 20–30 cm H_2_O), and a rise in HR. Ineffective ventilation was defined as not meeting one or more of the above criteria. A decrease in HR to <100 bpm from baseline indicated a lack of any manikin aeration and, therefore, poor performance. Low-pressure readings were presumed to represent a mask leak.

##### BMV Testing

After one month or more from baseline BMV assessment, participants underwent skills testing utilizing Scenario One to test skill retention. Participants did not have access to the trainer between baseline assessment and testing. Participants passed skills testing if they demonstrated effective BMV within two attempts. If more than two attempts were needed, skills testing was repeated every two or more weeks until participants passed. After passing Scenario One testing, participants then underwent more difficult testing utilizing Scenario Three (HR < 100 bpm and “low lung compliance”) before moving on to team training.

#### 2.2.3. Phase 3: Team Training

After completing Phase two, participants underwent a novel team training workshop with simulation practice. Prior to this study, teamwork was not a standard part of practice. All workshops contained a mixture of midwives and residents. Education covered: 1. pre-delivery focusing on equipment preparation, team assembly, role assignment and pre-briefing; 2. stabilization/resuscitation focusing on communication skills; and 3. post-stabilization focusing on debriefing and parent communication. Team members were encouraged to give verbal feedback on BMV performance (mask leak, chest rise, and HR changes) and to suggest two-person ventilation (one provider holding the mask with two hands and a second provider squeezing the bag) [12] if BMV was ineffective. Suctioning was deemphasized unless evidence of obstruction was found. Participants then completed one of four simulation scenarios. All simulation scenarios involved apneic infants requiring BMV. Each scenario was observed by the entire class and was followed by a class debrief. Workshop and simulation content were compiled by EA utilizing content from NRP [12] and Team STEPPS [13].

#### 2.2.4. Clinical Observations

Following completion of individual and team training, high-risk deliveries were intermittently observed by a research assistant (JC) from June 2024 to August 2025. Designation of a delivery as high-risk was made by the midwife and not JC. The aim was to evaluate adherence to the basic neonatal resuscitation steps including BMV when indicated. JC was an independent observer and not an active resuscitation member. Data collected included pre-delivery preparation, pre-briefing, team assembly, neonatal resuscitative steps, HR response to interventions, debriefing, and infant/parental interactions (skin-to-skin). Team assembly was defined as at least one midwife and resident being present prior to delivery. Observations were made on both the day and night shifts. Data was reviewed weekly with the research assistant and feedback was given to the study champions when indicated.

### 2.3. Data Collection and Analysis

All collected data was deidentified. Study data were collected and managed using REDCap electronic data capture tools hosted at Weill Cornell Medicine [14,15]. Surveys were completed electronically in REDCap or via paper and transcribed into REDCap. Data was analyzed with descriptive statistics, unpaired *t*-tests, Mann–Whitney U-tests, and chi-square analysis.

### 2.4. Ethics

This project was approved by the National Institute of Medical Research in Tanzania (NIMR/HQ/R.8a/Vol.IX/4566, approved 29 February 2024, renewed 25 June 2025) and received local institutional ethical approval (No. 2630, approved 8 May 2023). Participant involvement did not impact employment status. Verbal informed consent was obtained from the participants.

## 3. Results

The study comprised 16 midwives and 26 pediatric residents, of whom 100% of midwives and 96% of residents completed BMV testing, and 75% of midwives and 96% of residents underwent team training.

### 3.1. Phase 1—Pre-Assessment

Residents and midwives had similar knowledge scores (12.6 ± 1.8 versus 11.7 ± 2.1, *p* = 0.13). The majority of participants knew when to start BMV (midwives 100%; residents 92%), but responded incorrectly regarding effective BMV parameters. Most midwives responded incorrectly on the appropriate rate (75% incorrect), and both groups responded incorrectly on starting inflation pressure (midwives 50% incorrect; residents 69% incorrect). Midwives and residents had similar experiences of delivering BMV to an infant in the DR or on wards within the last six months (88% versus 81%, *p* = 0.57).

### 3.2. Phase 2—BMV Assessment and Testing

#### 3.2.1. BMV Baseline Assessment

Midwives, compared to residents, more often achieved all BMV parameters on the first attempt (88% vs. 27%, *p* < 0.01). Residents were more likely to ventilate with low inflating pressures (<20 mBar) (50% vs. 13%, *p* = 0.01). Poor ventilation performance, as reflected by a decrease in HR < 100 bpm, was noted in 12% of midwives and 38% of residents (*p* = 0.07, Table 1). With champion teaching, all participants were eventually able to demonstrate effective BMV after multiple attempts (a range of 1–4 attempts for both groups).

#### 3.2.2. BMV Testing—Scenario One

When comparing the baseline BMV assessment to Scenario One testing, effective BMV on the first attempt for midwives significantly decreased from 88% to 25% (*p* < 0.01), whereas residents improved (27% to 46%, *p* = 0.15). Residents took fewer attempts to achieve effective BMV parameters than midwives (*p* = 0.02, Table 1).

Common missed parameters for midwives included low (<20 mBar) (63%) or high (>30 mBar) (41%) inflating pressures and a slow rate (<40 bpm) (41%). A common missed parameter for residents was low inflation pressures (50%). Midwives were more likely than residents to utilize a slow ventilation rate (44% vs. 1%, *p* < 0.01) and have poor performance as evidenced by a HR drop <100 bpm (31% vs. 8%, *p* = 0.04) (Table 1).

Time between assessment and Scenario One testing was prolonged for both groups (midwives: 12.1 ± 2.9 weeks and residents: 10.1 ± 4.2 weeks, *p* = 0.09).

#### 3.2.3. BMV Testing—Scenario Three

Effective BMV on the first attempt for both midwives (50%) and residents (52%) was not significantly different on this more difficult scenario than from Scenario One. Midwives improved regarding the rate of ventilation breaths as fewer used a slow rate in Scenario Three than Scenario One (13% verses 44%, *p* = 0.04) (Table 1). Approximately one third of both midwives and residents used low inflation pressures.

### 3.3. Clinical Observations

#### 3.3.1. General Characteristics

After the team training workshop, 108 high-risk deliveries were observed by the research assistant from June 2024 to August 2025. These deliveries were attended by midwives ± residents. Infants were of birthweight 2.37 ± 1.03 kg and gestational age of 34.5 ± 3.7 weeks; 56% were male and 44% female. Most infants were delivered via c-section (73%). Common indications for a high-risk delivery were prematurity <37 weeks (65%) and pre-eclampsia/eclampsia (44%) (Table 2). Delayed cord clamping (≥1 min) occurred in 85% of neonates, including 9 of the 20 neonates who received BMV.

#### 3.3.2. Pre-Delivery

Pre-delivery, healthcare professionals always prepared equipment (100%), mostly performed a pre-brief (71%), and mostly assembled the team prior to delivery (71%) (Table 3). Failure for team assembly (*n* = 31) was secondary to precipitous delivery (29%), unknown reasons (29%), competing tasks on the wards (26%), residents not being called (10%), and residents not answering (6%).

#### 3.3.3. Stabilization

During all resuscitations, healthcare professionals promptly dried the infants, demonstrated teamwork, and did not utilize excessive suctioning (Table 3). Only seven (28%) infants <32 weeks of age were wrapped in plastic wrap as recommended by local hypothermia guidelines.

#### 3.3.4. Resuscitation

After initial drying, 27 (25%) infants were not crying, 7 of whom responded to stimulation techniques and 20 of whom required BMV. Of the infants who received BMV, 14 were performed by midwives and 6 were performed by residents. Two-person BMV was utilized for at least two cases. Fifteen cases were started within the golden minute (75%) (defined as within a minute of reaching the warmer). Effective BMV was demonstrated in 18 cases (90%), as evidenced by chest rises and a rise in HR > 100 bpm.

#### 3.3.5. Post-Stabilization

After stabilization, 80 (74%) neonates required admission to the special care nursery. Most were shown to their mother prior to admission (88%). Of those not admitted, 43% were placed skin-to-skin. General anesthesia was a common reason for lack of neonate–mother interaction. A debrief was conducted in only 9% of all deliveries (*n* = 108) and 25% of deliveries that required BMV (*n* = 20).

## 4. Discussion

This study highlights the complex nature of teaching neonatal resuscitation in the LRS and suggests the need for a multi-modal approach that includes both individual and team training. During individual manikin training, the midwives initially outperformed residents. However, this was not maintained on repeat testing, with midwives more often demonstrating poor BMV performance, as evidenced by a persistent HR < 100 bpm. Ineffective BMV was often secondary to low inflation pressures, which may represent a mask leak. Clinical observations demonstrated close adherence to pre-delivery preparation and basic neonatal stabilization/resuscitation interventions. Importantly, BMV appeared to be effective in most observed resuscitations as evidenced by chest rises and an increase in HR.

Pre-assessment surveys revealed that many first-line healthcare professionals understand the steps of resuscitation but not the correct parameters (rate and inflation pressure) to ensure BMV is effective. This likely reflects the absence of any pressure gauge on self-inflating bags often used in the LRS. Individual manikin training showed that objective feedback (Figure 2) with immediate repetitions can improve skills in the moment. This rapid cycle deliberate practice required dedicated champion input as multiple participants required several attempts to achieve effective BMV (Table 1).

Initially, more midwives demonstrated effective BMV within one attempt compared to residents. However, an unanticipated decline in skills amongst midwives was seen over repeated testing. Residents showed some improvement in Scenario One testing, but still only 50% were able to demonstrate effective BMV on the first attempt of subsequent testing. This suggests that rapid cycle deliberate practice does not prevent skill decay and thus frequent practice is likely necessary for the maintenance of skills, as described in previous studies [16,17,18]. We suspect that longer periods than intended between skill sessions may have contributed to the decline in skills. Gurung et al. demonstrated that eight skill drills with the NeoNatalie Live trainer over a three-month period were associated with maintenance of adequate ventilation amongst nurses in Nepal [17]. Additionally, Mduma et al. showed that brief weekly practice sessions coupled with monthly refresher trainings were associated with a decrease in early neonatal mortality [16].

A common issue during Phase 2 of the manikin training amongst both groups was low inflation pressures, which may represent a mask leak. These findings are similar to studies from resource-replete settings. Schilleman et al. found a large percentage of mask leaks when BMV was delivered to a manikin. Obstruction was also common as participants attempted to eliminate leaks [19]. Similarly, Schmölzer et al. found that mask leak occurred in 48% of cases and airway obstruction occurred in 25% of cases when BMV was administered to a preterm infant with a T-piece or self-inflating bag [20].

Based on the recommendations of NRP [12] and ILCOR [7], we implemented team training as an important addition to individual skill training. Team training included communication concepts (pre-briefs and closed-loop communication) and active feedback during a resuscitation. Team members were trained to report if there was chest rise and an increase in HR in response to ventilation and to transition to two-person ventilation if this was not present [12]. In support of this strategy, BMV was effective in 90% of apneic cases in the DR.

Team assembly is a vital component of efficient neonatal resuscitation. A list of indications was created for when team assembly was necessary. It was noted that ward tasks hindered resident DR attendance. Adjustments were made to the timing of when residents were called so they could better prioritize arriving prior to delivery.

Debriefing was the least adopted skill for unclear reasons. This possibly reflects both the learning process as well as the difficulties of team re-assembly after resuscitation. A scoping review conducted by ILCOR found two studies utilizing debrief checklists. One study found an increase in documentation and improvement in clinical outcomes. The second identified communication problems and improvement after consistent checklist use [21]. The introduction of a standardized checklist may improve adherence at KCMC.

### Limitations

This study has several limitations. First, there were no baseline clinical observational data, limiting the interpretation of some of the effects of training in relation to the effectiveness of BMV. Second, the duration between BMV scenarios may have affected skill retention. Third, in clinical practice, there was an inability to measure inflating pressure due to the use of self-inflating bags without pressure gauges. Fourth, initiation of BMV within the golden minute was defined as arrival to the warmer due to high rates of delayed cord clamping, instead of traditional definitions of within a minute of birth. Therefore, the likelihood of BMV being started within a minute of life is substantially less than the 75% observed. We suspect this is common in the LRS. Fifth, having an external observer may have influenced the care/decision-making of healthcare professionals, potentially biasing study results. Sixth, not all high-risk deliveries were observed, no baseline observations were made; and only a small number of neonates required BMV. Therefore, our findings should be interpreted with caution.

## 5. Conclusions

A multi-modal approach that combines individual BMV skills with team simulation training may enhance neonatal resuscitation in the LRS. Individual training revealed that front-line healthcare professionals recognize the need for BMV but are lacking in efficacy, which is most commonly secondary to low inflation pressures presumed in part to be related to mask leaks. While immediate feedback improved BMV technique, skill retention remained a challenge, underscoring the need for frequent structured practice sessions. The addition of team training, which has not been commonly reported in the LRS, appears to be associated with effective BMV in most observed cases. Future studies should focus on enhancing debriefing sessions.

## Figures and Tables

**Table 1:** BMV skills assessment and testing on the NeoNatalie Live trainer.

	Baseline		Scenario One		Scenario Three	
	Midwives (*n* = 16)	Residents (*n* = 26)	Midwives (*n* = 16)	Residents (*n* = 26)	Midwives (*n* = 16)	Residents (*n* = 25)
Effective BMV on First Attempt	14 (88%)	7 (27%) *	4 (25%)	12 (46%)	8 (50%)	13 (52%)
Median Attempts to Effective BMV (IQR)	1 (0)	2 (0.75)	4 (3.25)	2 (1) *	1.5 (1.25)	1 (1)
Range	1–4	1–4	1–9	1–5	1–7	1–3
Obstructed Head Positioning	1 (6%)	7 (27%)	2 (13%)	2 (8%)	1 (6%)	2 (8%)
Fast Rate	0 (0%)	2 (8%)	0 (0%)	4 (15%)	0 (0%)	1 (4%)
Slow Rate	1 (6%)	8 (31%)	7 (44%)	1 (4%) *	2 (13%)	0 (0%)
High Pressure	0 (0%)	1 (4%)	5 (31%)	0 (0%)	3 (19%)	3 (12%)
Low Pressure	2 (13%)	13 (50%) *	10 (63%)	13 (50%)	5 (32%)	8 (32%)
Heart Rate < 100 ^†^	2 (12%)	10 (38%)	5 (31%)	2 (8%) *	--	--

* Statistically significant differences between midwives and residents (*p* < 0.05). ^†^ Heart rate in Scenario Three starts below 100 bpm. Abbreviations: BMV = bag mask ventilation, IQR = Interquartile Range. Baseline and Scenario One represent an apneic infant with normal lung compliance and heart rate. Scenario Three represents an apneic infant with low lung compliance and a low initial heart rate.

**Table 2:** Indications for high-risk deliveries.

High-Risk Indication	N
Premature <37 weeks	70
Pre-eclampsia/eclampsia	48
Fetal distress	20
IUGR	11
GDMA	11
Placental abruption	8
Large baby	6
Oligohydramnios	6
Obstructed labor	4
Cord prolapse	2
Congenital anomaly	2
Fetal ascites	1

Abbreviations: IUGR = Intrauterine Growth Restriction, GDMA = Gestational Diabetes. Oligohydramnios is defined as amniotic fluid index <5 cm or single deepest pocket of <2 cm. Some deliveries have more than one indication.

**Table 3:** Post-workshop delivery room interventions for high-risk deliveries.

Pre-Delivery Preparations	*n* = 108
Equipment prepared	108 (100%)
Risk factors discussed	77 (71%)
Team assembled	77 (71%)
**Stabilization/Resuscitation**	
Dried Promptly	108 (100%)
Wet towel removed	107 (99%)
Plastic Wrap if <32 weeks	7/25 (28%)
Excessive suctioning	0 (0%)
Bulb suctioning	57 (53%)
Deep suctioning	3 (3%)
Required BMV	20 (19%)
Started in golden minute *	15/20 (75%)
Performed by midwife	14/20 (70%)
Performed by resident	6/20 (30%)
Chest rise or increase in HR	18/20 (90%)
Teamwork demonstrated	108 (100%)
**Post Stabilization Interventions**	
Infant admitted to special care nursery	80 (74%)
Infant shown to mother prior to NICU admission	70/80 (88%)
Placed skin to skin if not admitted	12/28 (43%)
Debrief occurred	9 (8%)

* Defined as starting within a minute of arriving at the warmer.

## Data Availability

Data is contained within the article or Supplementary Materials.

## Supplementary Materials

The following supporting information can be downloaded at https://www.mdpi.com/article/10.3390/children13050679/s1. Figure S1: Knowledge pre-tests administered to participants during Phase 1.

## Abbreviations

The following abbreviations are used in this manuscript:

AAP	American Academy of Pediatrics
BMV	Bag mask ventilation
HBB	Helping babies breathe
HR	Heart rate
ILCOR	International Liaison Committee on Resuscitation
KCMC	Kilimanjaro Christian Medical Centre
LRS	Low-resource setting
NRP	Neonatal Resuscitation Program
