# Supplementary material for: Individualized Skill-Based Manikin Training Coupled with a Team Approach May Enhance Delivery Room Neonatal Resuscitation in Low-Resource Settings
Source: Children (Basel). 2026 May 15;13(5):679. doi: 10.3390/children13050679 (PMC13204646; doi:10.3390/children13050679)
Supplement: Supplementary file 1 [file children-13-00679-s001.zip › children-4246120-supplementary.pdf]

Knowledge Pre-Test

1. What should you do in The Golden Minute?
   a. Bathe the baby
   b. Deliver the placenta
   c. Evaluate the heart rate
   d. Help a baby breathe if necessary

2. To prepare for a birth
   a. You identify a helper and review the emergency plan
   b. You ask everyone but the mother to leave the area
   c. You prepare equipment only when you need it
   d. You do not need a helper

3. To prepare the area for delivery
   a. Open all the doors and windows to get fresh air
   b. Darken the room
   c. Make sure the area is clean, warm, and well-lighted
   d. Keep the room temperature cold

4. What should you do to keep the baby warm?
   a. Open all the windows
   b. Give the baby a bath after birth
   c. Place hot water bottles next to the baby's skin
   d. Place the baby skin-to-skin with mother

5. Which baby can receive routine care after birth?
   a. A baby who is not breathing
   b. A baby who is gasping
   c. A baby who is crying/breathing well
   d. A baby who is limp

6. A baby is quiet, limp, and not breathing at birth. What should you do?
   a. Dry the baby thoroughly
   b. Shake the baby
   c. Throw cold water on the face
   d. Hold the baby upside down

7. A newborn baby is quiet, limp, and not breathing. The baby does not respond to steps in stimulate breathing. What should you do?
   a. Slap the baby's back
   b. Hold the baby upside down
   c. Squeeze the baby's ribs
   d. Begin ventilation

8. Suctioning a baby unnecessarily or frequently can:
   a. Cause a baby to stop breathing
   b. Make a baby start coughing and breathing
   c. Stimulate a baby to cry
   d. Increase the baby's heart rate

9. In which situation should a baby be suctioned?
   a. When a baby is crying at birth
   b. When a baby is crying but there is meconium in the amniotic fluid
   c. When a baby is not crying/breathing well and you see secretions blocking the mouth and nose
   d. Before drying the baby

10. Which of the following statements about ventilation with bag mask is TRUE?
    a. The mask should cover the eyes
    b. Air should escape between the mask and face
    c. Squeeze the bag to produce gentle movement of the chest
    d. Squeeze the bag to give 80 to 100 breaths per minute

11. A baby's chest is not moving with bag mask ventilation. What should you do?
    a. Stop ventilation
    b. Reapply the mask to get a better seal
    c. Clap the baby's back
    d. Give medicine to the baby

12. You can stop ventilation if
    a. A baby is blue and limp
    b. A baby's heart rate is slow
    c. A baby's heart rate is normal and the chest is not moving
    d. A baby's heart rate is normal and the baby is breathing or crying

13. At what heart rate can you stop giving bag mask ventilation if the baby is breathing on their own?
    a. <60 beats per minute
    b. 60-80 beats per minute
    c. 80-100 beats per minute
    d. >100 beats per minute

14. What rate should you deliver bag mask ventilation?
    a. 20-30 breaths per minute
    b. 30-40 breaths per minute
    c. 40-60 breaths per minute
    d. 60-80 breaths per minute

15. What inflating pressure should you start with when delivering bag mask ventilation?
    a. 10-20 cmH_2O
    b. 20-30 cmH_2O
    c. 30-40 cmH_2O
    d. 40-50 cmH_2O
